# Preoperative predictors of health-related quality of life changes (EQ-5D and EQ VAS) after total hip and knee replacement: a systematic review

**DOI:** 10.1186/s12891-021-04981-4

**Published:** 2022-01-17

**Authors:** Caroline Schatz, Nina Klein, Antonia Marx, Peter Buschner

**Affiliations:** 1 Ludwig-Maximilians-Universität München, LMU Munich School of Management, Institute of Health Economics and Health Care Management, Munich, Germany; 2 Helmholtz Zentrum München, Institute of Health Economics and Health Care Management, Munich, Germany; 3 Krankenhaus Barmherzige Brüder München, Akademisches Lehrkrankenhaus der Technischen Universität München, Munich, Germany

**Keywords:** PROM, Health-related quality of life, EQ-5D, Hip, Knee

## Abstract

**Background:**

Patient-reported outcomes are of ever-increasing importance in medical decision-making. The EQ-5D is one of the generic instruments measuring health-related quality of life (HRQoL) in arthroplasty. This review aimed to identify possible predictors of HRQoL changes for patients undergoing total knee replacements (TKR) or total hip replacements (THR).

**Methods:**

A systematic literature review according to the PRISMA guidelines was conducted, searching several databases. Preoperative to postoperative HRQoL changes were evaluated in patients undergoing THR or TKR, using the EQ-5D visual analog scale (VAS) or the preference-based EQ-5D Index were evaluated. Articles were considered with prospectively or retrospectively collected data, as well as registry data, each with statistical analyses of patient-related factors.

**Results:**

Eight hundred eighty-two articles were found, of which 21 studies met the inclusion criteria. Predictors were distinguished in alterable and non-alterable ones. The EQ-5D Index indicated a tendency towards beneficial improvements for patients with a high body mass index (BMI) (> 40) and no significant results for the VAS. Additionally, one study found that patient education and preoperative physiotherapy appeared to enhance HRQoL. Some evidence indicated that male gender was negatively associated with changes in the VAS and the EQ-5D Index, but one study reported the opposite. Changes in VAS and EQ-5D Index were lower for older patients, whereas a higher educational level seemed to be advantageous. A high Charnley class led to deteriorating changes in VAS, although a high Kellgren Lawrence classification was positively associated with the EQ-5D Index, in a limited number of studies. For all results, clinical relevance was calculated differently and mainly reported as uncertain or small.

**Conclusions:**

The literature on this topic was weak and offers only limited guidance. Results for alterable predictors, such as the BMI, indicated valuable improvements for highly obese patients. Further, high-quality research is required to support medical decision-making.

**Level of evidence:**

Level IV, according to the OCEBM Levels of Evidence Working Group.

**Supplementary Information:**

The online version contains supplementary material available at 10.1186/s12891-021-04981-4.

## Background

Total joint replacements are a recommended surgical procedure for patients with advanced osteoarthritis (OA). These interventions are performed to reduce pain and improve the function of joints for patients [1]. Despite the surgical success, there are patients who remain dissatisfied with total knee replacement (TKR) or total hip replacement (THR) [2, 3].

Patient-reported outcome measures are tools for the measurement of health-related quality of life (HRQoL). HRQoL mirrors the view of the individual patient and captures different aspects of this persons’ life, additionally to the perspective of the physician. According to Brooks (1996) [4] one of these measurements is the EQ-5D questionnaire, which quantifies the current state of health with a generic instrument. The questionnaire exists in two versions, the EQ-5D-3L and EQ-5D-5L, where the EQ-5D-5L is considered as the successor to the EQ-5D-3L [5, 6]. Both contain five dimensions (mobility, selfcare, usual activities, pain/discomfort, anxiety/depression) with each having three problem levels in the former and five in the latter. The vertical visual analog scale (VAS) completes the questionnaire. Patients evaluate their own, current health state by setting a mark between 0 being the worst health state and 100 being the best health state [4]. The psychometric properties of the EQ-5D were investigated for TKR and THR finding, among others, that the 5-L version showed improved performance [7]. Yet for THR, the EQ VAS scores were found to be highly correlated, and their estimates for different problem levels to be largely consistent between the 3 L and 5 L-versions [8–10].

A number of previous studies have examined predictors of postoperative HRQoL in patients undergoing TKR or THR. Studies have shown that mental health, especially pre-operative anxiety, depression, pain and poor function, predict a poorer post-operative HRQoL [11]. Socioeconomic status [12] or socioeconomic variables, such as age, gender and education were commonly used predictors for postoperative outcomes [13, 14], as well as obesity [15]. Additionally, preoperative function and preoperative radiological osteoarthritis seemed to be important predictors of postoperative HRQoL [16]. Newer approaches even used machine-leaning approaches to predict outcomes more precisely [17], or investigated predictors for disease-specific HRQoL instruments [12]. A detailed investigation about the predictive power of value sets, based on the EQ-5D-3L, showed that the preoperative surgery risk score, classified according to the American Society of Anesthesiologists (ASA), predicted HRQoL outcomes 1 year postoperatively [18].

This systematic review aimed to examine preoperative predictors for the improvement of preoperative to postoperative EQ-5D. The EQ-5D Index with the VAS is often applied in routine data collection pre/post THR and TKR procedures, for example in Sweden or the UK [18]. Since the EQ-5D with the VAS is a generic instrument, an analysis of predictors for improvement might identify alternative predictors to disease specific measurements such as the Western Ontario and McMaster Universities Osteoarthritis Index (WOMAC) [12].. The EQ-5D questionnaire also comprises questions about general health, especially psychological problems, that are often not considered in disease-specific measurements. Predicting the outcome of THR or TKR, in terms of HRQoL, remains a challenging but necessary task for researchers and practitioners. For this literature review, studies were selected that evaluated the improvement in HRQoL preoperatively to postoperatively, with predictors measured preoperatively. As some predictors, like age, were unchangeable and others like the BMI, might be changed, this review classified predictors as alterable and non-alterable. Additionally, a structural summary of the significance of predictors and investigation of the minimum clinical important difference (MCID) was provided, to contribute to the current discussion about HRQoL.

## Material and Methods

The method of this systematic literature review followed the PRISMA guidelines [19], with a narrative synthesis, without a meta-analysis. Variables in published studies, which improved HRQoL for patients with OA undergoing THR and TKR, were investigated. Beside the detection of predictors, the MCID was investigated, as well as the statistical significance. According to Page (2014) [20], especially in studies with patient-centered outcome measurements the clinical meaningfulness had to be considered, because the clinical insight was not adequately reflected by statistical significance. The *p*-value in statistics was chosen by the researcher and determines only a decision whether to decline the hypothesis or not, but this did not imply whether the change in HRQoL was meaningful for the patient [20]. The MCID was calculated for HRQoL instruments differently and an adaptation to the specific questionnaire was suggested [21]. A review revealed 11 methods to calculate the MCID for orthopedics [22]. Table 1 showed an excerpt of calculation methods for the MCID that were applied in the studies included in this review.

**Table 1:** Minimum clinical important differences (MCID)

Author	HRQoL	Indication	Outcome	Method
Walters et al. (2005) [23]	EQ-5D-3L, UK utility index	THR	Mean: 0,074 (−0.011 to 0.140)	Anchor-based (patient questions)
Impellizzeri et al. (2012) [24]	EQ-5D-3L, EQ VAS Score	Femoroacetabular impingement	≥ 15 points VAS	Anchor-based (patient questions)
Norman et al. (2003) [25]	HRQoL in general		Half of standard deviation	Distribution-based (review of 33 studies)
Cohen (1988) [26]	Effect size in general		0.20 (for a little effect)	Distribution-based (distance of means)

### Quality assessment

The quality of evidence was assessed according to the GRADE guidelines [27]. All included studies were examined by one author [CS] and at least a second author [NK, AM]. Disagreements were discussed among CS, NK, and AM leading to the finding of a collaborative solution.

The GRADE [27] assessment rated observational studies generally with low or very low quality. All studies in this review are observational and therefore, a low rating was the best assessment a study could achieve. According to Guyatt et al. (2011) [28], there are four further study limitations (inappropriate eligibility criteria, flawed outcome measurement, improper control of covariates, incomplete follow-up) that might increase the risk of bias in observational studies, and were considered in this review [29–32].. Details were provided in the supplementary material, as Additional file 1. All included studies developed transparent eligibility criteria and measured predictors and outcomes with an EQ-5D instrument. Covariates were controlled in several ways; mainly by alternating different variables in the statistical models. The statistical analyses were highly heterogenous with some studies including preoperative HRQoL in their model, whereas others used univariate analyses. Hence, collinearity could be an issue in some studies. The covariates for each study were provided in the supplementary material, as Additional file 2.

### Search strategy

**Fig. 1:**
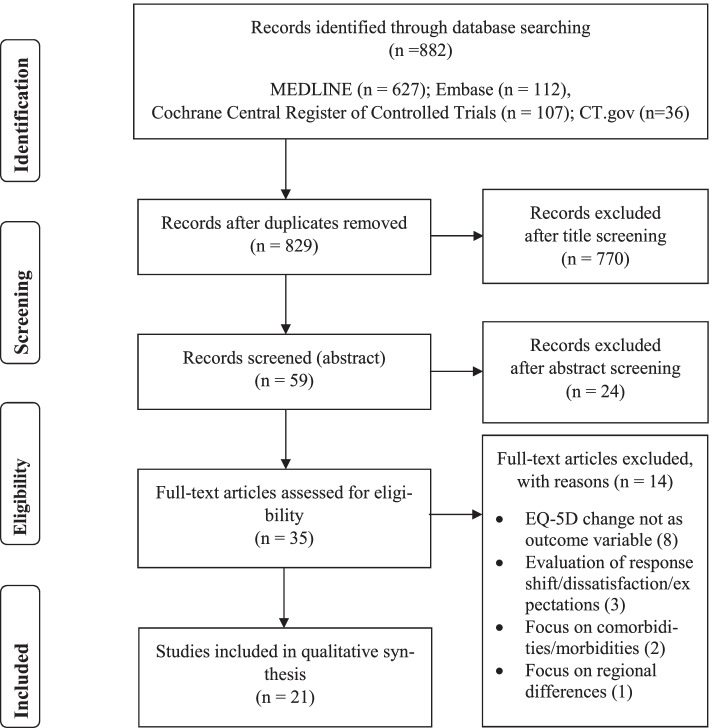
PRISMA flow diagram

The Cochrane Library and PubMed search engines were searched for articles until September 15, 2020 and updated on October 1, 2021. A detailed subdivision in the underlying databases was provided in Fig. 1. The PICO framework from the Cochrane Handbook [33] was applied, with the following search terms (Table 2).

**Table 2:** Search strategy with PICO

PICO	Search terms
Participants	hip OR knee
Interventions	artificial OR arthroplasty OR endoprosthesis OR prosthesis OR replacement
Comparisons	driver OR impact OR influence* OR factor OR predict* OR effect* OR importance OR change* OR shift OR improve* OR increase* OR trajector* OR difference OR gain*
Outcomes	EuroQol OR EQ-5D* OR EQ. 5D*

### Study selection

Inclusion criteria concerning the study design were randomized controlled trials (RCTs), trials, prospective studies and retrospective observational or register-based studies. These assessed predictive factors for HRQoL changes within OA patients who underwent primary TKR and THR. The HRQoL change was measured using the EQ-5D before and at least 3 months after the surgery with the original EQ-5D Index and VAS from the EuroQoL group [4]. To be included studies were required to assess the effect of no less than one preoperative predictive variable, but studies examining only individual comorbidities or multimorbidity were excluded. Studies were included if they were written in English or German languages.

### Data extraction

The software EndNote® was used to find duplicates and to screen titles and abstracts. Selected articles for full-text screening were examined using following, predefined examination criteria. Apart from the author, title and year of publication, the country, indication, joint, study design, method, time period, number of participants, response rate, applied level of questionnaire, and MCID were collected using Microsoft Excel® spreadsheets. A template was created and filled manually by CS, NK, and AM, from the studies. All studies were downloaded as pdfs and relevant text passages were marked digitally, with Acrobat Reader DC®. The explanatory variables were gathered and then distinguished as alterable or non-alterable ones. Further analysis was conducted regarding the effects of individual predictors on the EQ-5D Index and VAS scale with Microsoft Excel®.

## Results

Overall, 882 articles were found in both search engines. After duplicates were removed and titles and abstracts screened 35 articles were eligible. Of these, 14 articles were excluded, because the EQ-5D change was not the primary outcome variable. As a result, 21 articles were included in the qualitative analysis.

### Descriptive results

All 21 studies were conducted in the US or Europe. Mostly registry data were applied, either nation-wide or institution wide. Nation-wide, the Swedish Hip Arthroplasty Register was applied six times, the UK registry twice and the Dutch registry once. The number of patients varied widely from 147 up to 53.498, and the time period until the post-operative survey ranged from 3 months to 7 years, although several studies reported more than one postoperative response with different time periods. A wide variety of multiple regressions and tests were applied, as methods for the analysis (Table 3).

**Table 3:** Descriptive results

Author	Country	Joint (total)	Study design/data	Method	Time to follow-up	Number of patients
Baker et al. (2012) [34]	UK	Knee	National Joint Registry Data	Adjusted and unadjusted multiple regressions	6 to 12 months	13.673
Foster et al. (2015) [35]	USA	Hip	Institution-wide registry	F-tests	Not given	435
Galea et al. (2019) [36]	USA	Hip	Prospective, international, multi-center study	piece-wise linear mixed effects models	3 months, 1,3,5,7 years	976
Giesinger et al. (2021) [37]	Switzerland	Knee	Institution-wide study	Linear mixed models	12 months	1.565
Gordon et al. (2014) [38]	Sweden	Hip	Swedish Hip Arthroplasty Register	Robust covariance matrix	12 months	27.245
Greene et al. (2014) [39]	Sweden	Hip	Swedish Hip Arthroplasty Register	Linear regression models	12 months	11.464
Jenkins et al. (2013) [40]	UK	Hip/Knee	Regional registry	Two-way repeated measures analysis of variance	12 months	671
Joly et al. (2020) [41]	Canada	Hip/Knee	Retrospective study	descriptive analysis, one-way analysis of variance test, Chi-squared test, multivariate linear regression	3 and 12 months	53.498
Koekenbier et al. (2016) [42]	Finland, Greece, Iceland, Spain, Sweden	Hip/Knee	Prospective cohort study	General linear model	6 months	762
Manalo et al. (2018) [43]	USA	Knee	Institution-wide registry	Paired t-tests	12–17 months	167
McLawhorn et al. (2017) [44]	USA	Hip	Institution-wide registry	Pearson’s chi-square test, regression analysis	2 years	2.733
Mohaddes et al. (2019) [45]	Sweden	Hip	Swedish Hip Arthroplasty Register	Non-parametric tests	12 months	1.008
Ostendorf et al. (2004) [46]	USA	Knee	Institution-wide registry	Pair-wise comparisons using the Holm step-down Bonferroni method	2 years	147
Peters et al. (2020) [47]	Netherlands	Hip	Retrospective observational study, Dutch Arthroplasty Register	Multivariable linear regression analysis	3 and 12 months	22.357
Rehman et al. (2020) [48]	Norway	Knee	Longitudinal study	Paired sample t-tests, kappa statistics, Pearson correlation coefficients, multivariable regression analysis	12 months	245
Rolfson et al. (2011) [49]	Sweden	Hip	Swedish Hip Arthroplasty Register	Mann-Whitney U-test, multivariable regression	12 months	34.960
Scott et al. (2021) [50]	Scotland	Knee	Institution-wide study	Parametric and non-parametric tests, multivariable linear regressions	12 months	259
Steinhaus et al. (2020) [51]	UK	Knee	Institution-wide registry	Pair-wise comparisons using the Holm step-down Bonferroni method.	2 years	2.472
Tilbury et al. (2016) [52]	Netherlands	Hip Knee	Prospective cohort study	Mann-Whitney-U-test and multivariable linear regressions	12 months	573
Torisho et al. (2019) [53]	Sweden	Hip	Swedish Hip Arthroplasty Register	Multiple linear regression	12 months	30.756
Williams et al. (2013) [54]	UK	Knee (total/unicompartmental)	Prospective cohort study	Linear regression models	6 months	2.126

The results were subdivided into alterable predictors and non-alterable predictors. Alterable predictors encompassed the BMI, preoperative patient education, knowledge, and physiotherapy. Non-alterable predictors were defined as age, gender, level of education, Charnley class, Kellgren Lawrence (KL) classification, Ahlbäck classification, ASA-score/no previous operation and anxiety/depression Table 4.

**Table 4:** Summary results for alterable and non-alterable predictors

Predictor	Number of studies	EQ-5D Index results			EQ VAS results				MCID		
Alterable		**+**	**–**	**n.s.**	**+**	**–**	**n.s.**	**N.A.**	**+**	**–**	**N.A.**
High BMI	7	3	1	3	0	0	5	2	1	2	4
Opioid-user	1	0	0	1	0	0	1	0	0	0	1
Level of empowering knowledge	1	0	0	1	0	0	1	0	0	0	1
Patient education	1	1	0	0	1	0	0	0	0	0	1
Physiotherapy	1	1	0	0	1	0	0	0	0	0	1
Non-alterable											
High Charnley class	3	0	1	2	0	2	1	0	2	0	1
High KL classification	3	2	0	1	0	0	1	2	1	0	2
High Ahlbäck classification	1	0	0	1	0	0	0	0	0	0	1
Male gender	4	0	2	2	1	2	1	0	1	1	2
Oder age	8	0	4	4	0	4	3	1	2	1	5
High ASA score	1	1	0	0	0	0	1	0	0	1	0
Anxiety/depression	1	1	0	0	0	0	0	1	0	1	0
High education level	1	1	0	0	1	0	0	0	0	0	1
No previous operations	1	0	0	1	0	0	1	0	0	1	0

+ positive relationship, − negative relationship, *n.s* not significant, *N.A* not applicable

### Alterable predictors

#### BMI

The influence of the BMI on HRQoL changes was investigated for TKR by Baker et al. (2012) [34], finding EQ-5D Index changes to increase with a higher preoperative BMI, subdivided into three groups (BMI of 15–24; 25–39; 40–60). After adjusting for age, gender, ASA grade, comorbidities and health ratings, no significant impact of any BMI group was found on the EQ-5D Index change. Also, the results were not considered to be clinically relevant, and the VAS scores were not significant, irrespective of the adjustments and the BMI group [34]. Partly in contrast, Steinhaus et al. (2020) [51] investigated no significant differences in VAS changes between different BMI groups, measured according to the WHO definition [55], but for the EQ-5D Index. Equally, for the EQ-5D Index, Giesinger et al. (2021) [37] found no significant results on the BMI classes for TKR, considering age, gender and the interaction of time point (preoperative and 12-month follow-up) by BMI group, as covariates. Patients from all BMI groups experienced HRQoL improvements and patients with a BMI in obesity classes II and III experienced the largest EQ-5D Index improvements [37, 44, 51].

THR was evaluated by McLawhorn et al. (2017) [44]: for the VAS no significant impact of the BMI, according to the WHO classification [55], was revealed. However, for the EQ-5D Index, obese patients significantly improved their HRQoL by THR, especially patients with a BMI > 40 and underweight patients with a BMI < 18,5 [44]. In contrast, Foster et al. (2015) [35] also evaluated the impact of BMI, with three subgroups (< 30; 30–40; > 40), on HRQoL changes and found no significant influence for the VAS or the EQ-5D Index changes [35]. Peters et al. (2020) [47] examined the influence of the EQ-5D Index on the BMI, distinguished between lower than 30 and above 30. It was confirmed that patients with a high BMI benefit more from THR, but with a small Cohen’s *d*, indicating small clinical relevance [47]. It is important to mention that Peters et al. (2020) [47] and Foster et al. (2015) [35] did not evaluate underweight patients in a separate BMI class. Using long-term results after 1 year, Galea et al. (2019) [36] showed that obese patients (BMI ≥30) steadily declined in the EQ-5D Index after an increase in HRQoL in the first 3 months after THR.

#### Preoperative patient education, knowledge, and physiotherapy

A cross-cultural study by Koekenbier et al. (2016) [42] evaluated the influence of expected and received patient education on HRQoL changes. The hypothesis, that patients, who received their expected amount of patient education, would more likely be empowered and therefore able to manage their condition, leading to a higher HRQoL, was investigated. There was no association between the level of empowering knowledge and the improvement in HRQoL, neither for the EQ-5D Index nor for the VAS. The countrywide comparison showed that higher levels of empowering knowledge had a significant positive influence on the VAS improvement only in Greece [42]. Torisho et al. (2019) [53] examined patient education before THR and found minor positive associations with an improvement in EQ-5D Index and VAS. Patient education was enabled by the Supported Osteoarthritis Self-Management Program, where participants were guided into groups, and therapy was adapted individually. Physiotherapy was a part of this program, in addition to patient education. A positive relationship between improvements in HRQoL and physiotherapy for the EQ-5D Index and VAS was revealed, but also with uncertain clinical relevance [53].

#### Opioid usage

Manalo et al. (2018) [43] evaluated the impact of preoperative opioid medication use on HRQoL changes after TKR. There was a significant HRQoL improvement for non-opioid users and no significant HRQoL improvement for opioid users on the EQ-5D Index and VAS. Even though the first finding indicated that non-opioid users benefit more from the surgery, no significant difference was found in the EQ-5D Index and VAS improvements between the opioid and non-opioid group [43]. Additional file 3 in the supplementary material provides detailed information about all alterable predictors.

### Non-Alterable Predictors

#### Socioeconomic and demographic variables

##### Age and gender

The influence of age and gender on the EQ-5D Index and VAS in TKR and THR was evaluated by Jenkins et al. (2013) [40] with conflicting results. Although male gender was significantly associated with an increased VAS improvement, there was no impact on the EQ-5D Index change, whereas age had no significant impact on the VAS change, or on the EQ-5D Index. Generally, THR showed greater improvements than TKR [40]. THR was investigated by Peters et al. (2020) [47], and age and gender were regarded as being associated with an improvement in VAS and the EQ-5D Index. Women and patients < 60 years benefited more from THR than men and older patients [47]. These findings were in line with the study from Rolfson et al. (2011) [49] who reported lower improvements for both, male gender and older age (> 60 years) for VAS and EQ-5D Index, equally for THR. Conversely, Foster et al. (2015) [35] found no significant effects for either gender or age (< 65 years).

##### Age

Gordon et al. (2014) [38] investigated the influence of age on HRQoL in THR. Age was divided into 6 age groups (≤ 50, 51–60, 61–70, 71–80, 81–85 and > 85 years). Even though, improvements were found in VAS and the EQ-5D Index across all age groups, 20% of patients older than 80 years were not able to improve their VAS score and 13% of patients older than 80 years did not improve their EQ-5D Index. Therefore, a non-linear significant negative impact of age on VAS and EQ-5D Index improvements was revealed, starting in the late sixties. Additionally, low preoperative HRQoL had a significant effect on the EQ-5D improvements, with lower preoperative values resulting in larger gains [38]. Equally for THR, but for young patients (< 30 years) with OA or inflammatory joint disease, Mohaddes et al. (2019) [45] found significant VAS improvements compared to patients older than 30 years, whereas no significant impact was reported on the EQ-5D Index [45].

For TKR and unicompartmental knee replacements Williams et al. (2013) [54] indicated equally unbalanced results for the impact of age on HRQoL improvements. Patients were grouped into 5 age groups (< 55, 55–64, 54–74, 75–84 and ≥ 85 years) and a significant linear impact was investigated for the EQ-5D Index with higher age resulting in lower EQ-5D Index changes. For the VAS, no significant influence of age was reported [54]. Joly et al. (2020) [41] indicated lower improvements for EQ-5D scores in younger patients (< 55 years) at 3 months postoperatively for TKR compared to patients aged > 70 years. There was no significant difference at 1 year postoperatively. In contrast, for THR, the EQ-5D scores improved in younger patients (< 55 years) after 1 year, and the improvements were fewer after 3 months [41].

##### Level of education

The educational level was examined by Greene et al. (2014) [39] as a predictor of HRQoL for THR. Patients with no education beyond primary school were assigned to the low education level, patients with no education beyond secondary school to the medium education level and patients with any postsecondary education to the high education level. The results indicated that patients with higher education attainment levels had significantly greater EQ-5D Index and VAS improvements in comparison to patients with lower or medium education attainment [39].

#### Severity

##### Charnley class

Rolfson et al. (2011) [49], analyzed the influence of gender, age, and Charnley class on HRQoL changes for THR. Charnley class was revealed as a strong predictor for HRQoL measured with the VAS and the EQ-5D Index. Less improvement was reported, compared with a reference population, especially for class C [49]. In a similar way, Ostendorf et al. (2004) [46] evaluated the impact of Charnley classes on HRQoL changes for THR. The effect size was calculated by dividing the difference in pre- and postoperative EQ-5D scores by the preoperative score standard deviation. Medium effect sizes were found for the change in VAS for patients in class A and small effect sizes for patients in classes B and C. For the EQ-5D Index, a large degree of change was reported for patients in all classes. Patients in class A improved significantly more than patients in classes B and C, measured with the VAS. There were no significant differences between pre- and postoperative EQ-5D scores for the different classes [46]. Conversely, Foster et al. (2015) [35] investigated the impact of Charnley classes on the EQ-5D Index and VAS and found no significant differences between pre- and postoperative scores.

##### Kellgren Lawrence classification (KL)

The KL score is a classification system for OA. The influence of KL on HRQoL improvements by TKR and THR were examined by Tilbury et al. (2016) [52]. Differences in the HRQoL changes of patients with KL grades 0–2 in comparison to patients with KL grades 3–4 were evaluated. THR patients with a KL grade 3–4 showed greater HRQoL improvement than patients with a KL grade of 0–2, although this result was only significant for the EQ-5D Index and not for the VAS. Results for TKR patients were not significant. Adjusted for age, gender, and BMI, as well as Charnley class for the THR group, no significant effects of KL grade on HRQoL changes were investigated, for either TKR or THR was investigated [52]. Scott et al. (2021) [50] also found no significant effects of the KL grade on HRQoL changes after TKR. In contrast, a study by Rehman et al. (2020) [48] evaluated whether severe radiographic OA was related to improvements in HRQoL after TKR. The results indicated that patients with severe OA, especially KL grade 4, had more clinically meaningful improvement in their EQ-5D Index than patients with less severe OA [48].

##### Ahlbäck classification

As well as the KL classification Scott et al. (2021) [50] investigated the Ahlbäck classification. Similar to the KL classification, they found no significant effect of the Ahlbäck classification on the improvement in the EQ-5D Index after TKR.

##### ASA score/no previous operations

Peters et al. (2020) [47] examined the influence of the ASA score on the VAS and the EQ-5D Index for THA. The results showed diverging effects, with an improvement for patients with a high ASA score (III-IV) for the EQ-5D Index, but a non-significant result for the VAS. The clinical relevance was regarded to be very small. No influence of a previous operation was found, for either the EQ-5D Index, or the VAS [47].

##### Anxiety/depression

Anxiety/depression as one item in the EQ-5D-3L was evaluated as a predictor for the EQ-5D Index by Galea et al. (2019) [36] following THR. One year postoperatively, smaller improvements were found in comparison to the rest of the cohort [36].

Detailed information about non-alterable predictors was given in the supplementary material, Additional file 4. The following table summarized the analyzed predictors for alterable and non-alterable predictors (Table 3).

## Discussion

The evidence for preoperative predictors for changes in the EQ-5D Index or VAS was unclear. The most important main finding of this review was the effect that patients with higher BMI appeared to have increased improvements in their EQ-5D Index, preoperatively to postoperatively, especially for patients with a BMI > 40. This finding should be weighed against the higher general- and specific risks of joint replacement surgeries for obese patients [56]. One study found that the delivery of patient education and physiotherapy was associated with better EQ-5D scores [53]. Significant results for non-alterable factors showed a tendency towards declining improvements for male gender (2 studies) [47, 49], older patients (5 studies) [38, 47, 49, 54], conflicting results for severity (4 studies) [47–49, 52] and benefits for higher education (1 study) [39]. The effects of preoperative predictors seemed not to be understood. Although, as mentioned by Rolfson et al. (2011) [49], differences between pre- and postoperative EQ-5D distributions might prevent significant effects. In particular, comparing preoperative heterogeneous patient groups with postoperative homogeneous patient groups could prevent the identification of significant preoperative predictors. Furthermore, patients might be selected by hospitals and physicians, balancing high-risk patients with low-risk patients. This might have influenced regression results toward non-significant outcomes.

Considering comparison with MCIDs, only one study indicates clinical relevance, reporting the reverse effect for a higher BMI [36]. A limitation given different concepts of the MCID, and a range of values found, the MCID used was mostly not reported, but there were studies showing clinically relevant results regarding male gender, older age and severity. These findings were partly in line with previous reviews on predictors influencing postoperative HRQoL. For THR, determining preoperative factors that influence postoperative outcomes seemed to be very difficult, with vague results indicating that improvements were higher for worse preoperative function and more severe radiological OA [16]. Predictors for TKR, measured with the WOMAC, indicated ambiguous results, too. Older patients might benefit more from TKR than younger patients and female gender seemed to be advantageous, but there might also be no difference. The socioeconomic status had been reported to be beneficial [12], which was in accordance with this review. Very weak and no clear association for a variety of preoperative factors, like BMI, gender, comorbidities and preoperative function were found for TKR, measured with disease-specific HRQoL tools [13]. Predictors for functional outcomes were investigated by Buirs et al. (2016) [57] with diverse HRQoL instruments. Negative associations with high BMI and higher age were reported, whereas the association with good mental health seemed to be positive, and the impact on gender ambiguous. Chesham et al. (2017) [58] investigated whether preoperative physiotherapy improved the outcome of TKR. They found that more, high-quality, research was needed to draw a succinct conclusion. Thus, these mainly disease-specific literature reviews indicated a controversial discussion about predictors of HRQoL for THR and TKR. With the investigation of the generic instrument of the EQ-5D in this review, the controversies remain. The alterable predictors may be used by clinicians to improve the postoperative outcome of patients by taking preoperative measures.

## Limitations

All studies were observational, in part lacking confounders, and mainly derived from routine data. The bias across studies might be strong, because the comparisons were made upon some simplifications. This review made no exclusion for the follow-up time after surgery. Hence, it would be possible that significant results differ for a follow-up time of 3 months compared with several years. Additionally, 12 studies applied the EQ-5D-3L, whereas others used the EQ-5D-5L or gave no information about the level. Although psychometric studies showed improvements in HRQoL measurement of the 5 L-version over the 3 L-version of the EQ-5D, this still cannot fully explain the opposite effects on HRQoL changes found for some of the predictors, for example the BMI [36].

Regarding the study design, the main database was the MEDLINE, there may be articles in additional databases that were not considered in this review. All articles were equally weighted. Some authors contributed to several articles with similar data and might not contradict themselves. Registry data were also applied by the same author group (e.g., Swedish registry data), which might cause bias towards these results.

Moreover, the MCID was reported in only 8 studies, with a calculation of the clinical relevance in several ways. The statistical effect size of Cohen’s d [26] (0.2) and the anchor-based threshold of Walters et al. (2005) [23] (0.074) were mostly applied in slightly different versions and combinations. Owing to the heterogeneous thresholds, it remained unclear whether the changes in HRQoL were relevant. In future work, validation of the MCIDs used is thus highly important, with the respective strategies recently developed [59]. Furthermore, the response rates and the number of participants varied widely. The comparison was additionally impaired, because several statistical methods were applied.

## Conclusion

There was limited evidence for definitive, preoperative predictors of HRQoL for TKR and THR. Alterable factors were mainly not significant, measured with the VAS. Higher BMI was associated with greater improvements in the EQ-5D Index. This was contradictory to the higher general- and specific risk of a joint surgery for obese patients, as reported by Kerkhoffs et al. (2012) [56] for infections and revisions in TKR. For non-alterable factors, male gender, older age and high Charnley class seemed to be negatively associated with HRQoL gains and thus pointed to additional potential risks, whereas KL and higher education appeared to be advantageous. The MCID was regarded to be uncertain. All the studies were observational and 42% of them were of very low quality. Higher quality studies are needed to identify predictors of change in EQ-5D Index and VAS following TKR and THR. This knowledge would support the optimal preoperative preparation of patients and assist with shared decision-making for arthroplasty. Further research is needed to support optimal preoperative preparation of patients as well as to provide better information for shared decision-making on arthroplasty.

## Supplementary Information

**Additional file 1.**

**Additional file 2.**

**Additional file 3.**

**Additional file 4.**

## Data Availability

Not applicable.

## Abbreviations

ASA: American Society of Anesthesiologists
BMI: Body Mass Index
VAS: EuroQoL visual analog scale
HRQoL: Health-Related Quality of life
KL: Kellgren Lawrence classification
MCID: Minimum Clinical Important Difference
OA: Osteoarthritis
THR: Total Hip Replacement
TKR: Total Knee Replacement
WOMAC: Western Ontario and McMaster Universities Osteoarthritis Index
