# Supplementary material for: Preoperative predictors of health-related quality of life changes (EQ-5D and EQ VAS) after total hip and knee replacement: a systematic review
Source: BMC Musculoskelet Disord. 2022 Jan 17;23:58. doi: 10.1186/s12891-021-04981-4 (PMC8764845; doi:10.1186/s12891-021-04981-4)
Supplement: Supplementary file 1 — Additional file 1. [file 12891_2021_4981_MOESM1_ESM.docx]

**Additional file 1** Quality assessment according to GRADE [27]

| Author | Title | Study design | Study  limitations | Inconsistency | Indirectness | Imprecision | Publication bias | Quality |
| --- | --- | --- | --- | --- | --- | --- | --- | --- |
| *Evaluation criteria* | | *RCT –*  *very high, high*  *Observational –*  *low, very low* | *Response rate,*  *loss to follow-up* | *Unexplained heterogeneity* | *Direct measurement of evidence* | *Low number of participants, high confidence intervals* | *Data quality* | *Overall rating* |
| Baker et al. (2012) [34] | The association between body mass Index and the outcomes of total knee arthroplasty. | Observational study | Serious | No serious inconsistency | No serious indirectness | No serious imprecision | Undetected | Low |
| Foster et al. (2015) [39] | Effects of Obesity on Health Related Quality of Life Following Total Hip Arthroplasty. | Observational study | Very serious | No serious inconsistency | No serious indirectness | No serious imprecision | Undetected | Very low |
| Galea et al. (2019) [41] | Longitudinal changes in patient-reported outcome measures following total hip arthroplasty and predictors of deterioration during follow-up: a seven-year prospective international multicentre study | Observational study | Serious | Serious inconsistency | No serious indirectness | No serious imprecision | Strongly suspected | Very low |
| Giesinger et al. (2021) [37] | Higher body mass index is associated with larger postoperative improvement in patient-reported outcomes following total knee arthroplasty. | Observational study | Serious | No serious inconsistency | No serious indirectness | No serious imprecision | Undetected | Low |
| Gordon et al. (2014) [47] | Age- and health-related quality of life after total hip replacement: decreasing gains in patients above 70 years of age. | Observational study | Serious | No serious inconsistency | No serious indirectness | No serious imprecision | Undetected | Low |
| Greene et al. (2014) [51] | Education attainment is associated with patient-reported outcomes: findings from the Swedish Hip Arthroplasty Register. | Observational study | Serious | No serious inconsistency | No serious indirectness | No serious imprecision | Undetected | Low |
| Jenkins et al. (2013) [45] | Predicting the cost-effectiveness of total hip and knee replacement: a health economic analysis. | Observational study | Serious | No serious inconsistency | No serious indirectness | No serious imprecision | Undetected | Low |
| Joly et al. (2020) [50] | Does Age Influence Patient-Reported Outcomes in Unilateral Primary Total Hip and Knee Arthroplasty? | Observational study | Serious | No serious inconsistency | No serious indirectness | No serious imprecision | Undetected | Low |
| Koekenbier et al. (2016) [42] | Empowering knowledge and its connection to health-related quality of life: A cross-cultural study. | Observational study | Very serious | No serious inconsistency | No serious indirectness | No serious imprecision | Undetected | Very low |
| Manalo et al. (2018) [44] | Preoperative opioid medication use negatively affect health related quality of life after total knee arthroplasty. | Observational study | Very serious | Serious inconsistency | No serious indirectness | Serious imprecision | Strongly suspected | Very low |
| McLawhorn et al. (2017) [38] | Body Mass Index Class Is Independently Associated With Health-Related Quality of Life After Primary Total Hip Arthroplasty: An Institutional Registry-Based Study. | Observational study | Serious | No serious inconsistency | No serious indirectness | Serious imprecision | Undetected | Very low |
| Mohaddes et al. (2019) [48] | Implant survival and patient-reported outcome following total hip arthroplasty in patients 30 years or younger: a matched cohort study of 1,008 patients in the Swedish Hip Arthroplasty Register | Observational study | Serious | No serious inconsistency | No serious indirectness | No serious imprecision | Undetected | Low |
| Ostendorf et al. (2004) [52] | Body Mass Index Classification Is Independently Associated with Health-Related Quality of Life after Primary Total Knee Arthroplasty: An Institutional Registry-Based Study. | Observational study | Serious | No serious inconsistency | No serious indirectness | No serious imprecision | Strongly suspected | Very low |
| Peters et al. (2020) [40] | Which patients improve most after total hip arthroplasty? Influence of patient characteristics on patient-reported outcome measures of 22,357 total hip arthroplasties in the Dutch Arthroplasty Register | Observational study | Serious | No serious inconsistency | No serious indirectness | No serious imprecision | Undetected | Low |
| Rehman et al. (2020) [55] | More Severe Radiographic Osteoarthritis Is Associated With Increased Improvement in Patients' Health State Following a Total Knee Arthroplasty | Observational study | Serious | No serious inconsistency | No serious indirectness | Serious imprecision | Strongly suspected | Very low |
| Rolfson et al. (2011) [46] | Patient-reported outcomes in the Swedish Hip Arthroplasty Register: results of a nationwide prospective observational study. | Observational study | Serious | No serious inconsistency | No serious indirectness | No serious imprecision | Undetected | Low |
| Scott et al. (2021) [54] | Radiographic severity, extent and pattern of cartilage loss are not associated with patient reported outcomes before or after total knee arthroplasty in end-stage knee osteoarthritis | Observational study | Serious | No serious inconsistency | No serious indirectness | No serious imprecision | Undetected | Low |
| Steinhaus et al. (2020) [35] | Body Mass Index Classification Is Independently Associated with Health-Related Quality of Life after Primary Total Knee Arthroplasty: An Institutional Registry-Based Study. | Observational study | Serious | No serious inconsistency | No serious indirectness | No serious imprecision | Undetected | Low |
| Tilbury et al. (2016) [53] | Outcome of total hip arthroplasty, but not of total knee arthroplasty, is related to the preoperative radiographic severity of osteoarthritis. A prospective cohort study of 573 patients. | Observational study | Very serious | No serious inconsistency | No serious indirectness | No serious imprecision | Undetected | Very low |
| Torisho et al. (2019) [43] | Minor influence of patient education and physiotherapy interventions before total hip replacement on patient-reported outcomes: an observational study of 30,756 patients in the Swedish Hip Arthroplasty Register | Observational study | Serious | No serious inconsistency | No serious indirectness | No serious imprecision | Undetected | Low |
| Williams et al. (2013) [49] | The effects of age on patient-reported outcome measures in total knee replacements. | Observational study | Serious | No serious inconsistency | No serious indirectness | No serious imprecision | Undetected | Low |
