# Supplementary material for: Preoperative predictors of health-related quality of life changes (EQ-5D and EQ VAS) after total hip and knee replacement: a systematic review
Source: BMC Musculoskelet Disord. 2022 Jan 17;23:58. doi: 10.1186/s12891-021-04981-4 (PMC8764845; doi:10.1186/s12891-021-04981-4)
Supplement: Supplementary file 2 — Additional file 2. [file 12891_2021_4981_MOESM2_ESM.docx]

Additional file 2 **Covariates and variables of interest for each study.**

| Author | All variables | | | | | Variable of  interest |
| --- | --- | --- | --- | --- | --- | --- |
|  | **Age** | **Gender** | **ASA** | **BMI** | **Other** |  |
| Baker et al. (2012) [34] | x | x | x | x | number of comorbidities, general health rating | BMI |
| Foster et al. (2015) [39] | x | x |  | x | Charnley class | BMI, Charnley, Gender, Age |
| Galea et al. (2019) [41] | x | x |  | x | Mental health (subscore SF-36) | BMI, anxiety |
| Giesinger et al. (2021) [37] |  |  |  | x | time point, interaction BMI and time point | BMI |
| Gordon et al. (2014) [47] | x | x |  |  | preoperative HRQoL, previous contralateral hip surgery, pain | Age |
| Greene et al. (2014) [51] | x | x |  |  | educational level, Charnley class, comorbidity,  preoperative HRQoL, Hip order, marital status | Educational level |
| Joly et al. (2020) [50] | x | x |  |  | presurgery risk factors | Age |
| Jenkins et al. (2013) [45] | x | x |  |  | Comorbidities, TKR versus THR, preoperative HRQoL | Gender, Age |
| Koekenbier et al. (2016) [42] |  |  |  |  | expected and received knowledge | Empowering knowledge |
| Manalo et al. (2018) [44] |  |  |  |  | Opiod | Opiod |
| McLawhorn et al. (2017) [38] | x | x |  | x | laterality, year of surgery, Charlson comorbidity index, length of stay | BMI |
| Mohaddes et al. (2019) [48] | x |  |  |  |  | Age |
| Ostendorf et al. (2004) [52] |  |  |  |  | Charnley class | Charnley class |
| Peters et al. (2020) [40] | x | x | x | x | Charnley class, smoking, previous operations, PROMs | BMI, Gender, Age, ASA score, no previous operation |
| Rehman et al. (2020) [55] | x | x | x | x | Number of comorbidities, prior ipsilateral knee surgery, prior arthroplasty, preoperative worst pain rating | KL classification |
| Rolfson et al. (2011) [46] | x | x |  |  | Diagnoses, Charnley class | Charnley, Gender, Age |
| Scott et al. (2021) [54] | x | x |  | x | KL classification, Ahlbäck class | KL classification, Ahlbäck class |
| Steinhaus et al. (2020) [35] | x | x |  | x | laterality, year of surgery, Charlson-Deyo comorbidity index | BMI |
| Torisho et al. (2019) [43] | x | x | x | x | Charnley class, incision, fixation, patient education, physiotherapy | Patient education, Physiotherapy |
| Tilbury et al. (2016) [53] | x | x |  | x | level of education, marital status, job, Charnley class | KL classification |
| Williams et al. (2013) [49] | x | x | x | x | Implant differences | Age |

x variable was included in the model
