# Supplementary material for: Preoperative predictors of health-related quality of life changes (EQ-5D and EQ VAS) after total hip and knee replacement: a systematic review
Source: BMC Musculoskelet Disord. 2022 Jan 17;23:58. doi: 10.1186/s12891-021-04981-4 (PMC8764845; doi:10.1186/s12891-021-04981-4)
Supplement: Supplementary file 3 — Additional file 3. [file 12891_2021_4981_MOESM3_ESM.docx]

**Additional file 3: Alterable predictors**

| Autor | Predictor | Index | VAS | MCID |
| --- | --- | --- | --- | --- |
| Baker et al. (2012) [34] | High BMI | n.s. | n.s. | - ^a^ |
| Foster et al. (2015) [39] | High BMI | n.s. | n.s. | N.A. |
| McLawhorn et al. (2017) [38] | High BMI | + | n.s. | N.A. |
| Steinhaus et al. (2020) [35] | High BMI | + | n.s. | N.A. |
| Galea et al. (2019) [41] | High BMI | -  (1-7 years) | N.A. | + ^b^ |
| Peters et al. (2020) [40] | High BMI | + | n.s. (1 year) | - ^c^ |
| Giesinger et al. (2021) [37] | High BMI | n.s. | N.A. | N.A. |
| Manalo et al. (2018) [44] | Opioid-user | n.s. | n.s. | - ^d^ |
| Koekenbier et al. (2016) [42] | Level of empowering  knowledge | n.s. | n.s. | N.A. |
| Torisho et al. (2019) [43] | Patient education | + | + | N.A. |
| Torisho et al. (2019) [43] | Physiotherapy | + | + | N.A. |

^a^ Method not specified; ^b^ Half of standard deviation; ^c^ Cohen (1988) [26]; ^d^ Impellizzeri et al. (2012) [24], Cohen (1988) [26]; n.s. not significant; N.A. not applicable; + positive correlation; - negative correlation
