# Supplementary material for: Preoperative predictors of health-related quality of life changes (EQ-5D and EQ VAS) after total hip and knee replacement: a systematic review
Source: BMC Musculoskelet Disord. 2022 Jan 17;23:58. doi: 10.1186/s12891-021-04981-4 (PMC8764845; doi:10.1186/s12891-021-04981-4)
Supplement: Supplementary file 4 — Additional file 4. [file 12891_2021_4981_MOESM4_ESM.docx]

**Additional file 4: Non-alterable predictors**

| Author | Predictor | Index | VAS | MCID |
| --- | --- | --- | --- | --- |
| Foster et al. (2015) [39] | High Charnley class | n.s. | n.s. | N.A. |
| Rolfson et al. (2011) [46] | High Charnley class | - | - | + ^a^ |
| Ostendorf et al. (2004) [52] | High Charnley class | n.s. | - | + ^b^ |
| Tilbury et al. (2016) [53] | High KL classification | n.s. (TKR)  + (THR) | n.s. | N.A. |
| Rehman et al. (2020) [55] | High KL classification | + | N.A. | + ^a^ |
| Scott et al. (2021) [54] | High KL classification  High Ahlbäck classification | n.s. | N.A. | N.A. |
| Foster et al. (2015) [39] | Male gender | n.s. | n.s. | N.A. |
| Jenkins et al. (2013) [45] | Male gender | n.s. | + | N.A. |
| Rolfson et al. (2011) [46] | Male gender | - | - | + ^a^ |
| Peters et al. (2020) [40] | Male gender | - | - | - ^c^ |
| Foster et al. (2015) [39] | Older age | n.s. | n.s. | N.A. |
| Mohaddes et al. (2019) [48] | Older age | n.s. | - | N.A. |
| Gordon et al. (2014) [47] | Older age | - | - | N.A. |
| Jenkins et al. (2013) [45] | Older age | n.s. | n.s. | N.A. |
| Rolfson et al. (2011) [46] | Older age | - | - | + ^a^ |
| Williams et al. (2013) [49] | Older age | - | n.s. | + ^a^ |
| Joly et al. (2020) [50] | Older age | n.s. (>1 year) | N.A. | N.A. |
| Peters et al. (2020) [40] | Older age | - | - | - ^b^ |
| Peters et al. (2020) [40] | High ASA score | + | n.s. | - ^b^ |
| Galea et al. (2019) [41] | Anxiety/depression | -  (1 year) | N.A. | - ^c^ |
| Greene et al. (2014) [51] | High education level | + | + | N.A. |
| Peters et al. (2020) [40] | No previous operations | n.s. | n.s. | - ^b^ |

^a^ Walters et al. (2005) [23]; ^b^ Cohen (1988) [26]; ^c^ Half of standard deviation; n.s. not significant; N.A. not applicable; + positive correlation; - negative correlation
